# Dynamics of Bacterial Root Endophytes of *Malus domestica* Plants Grown in Field Soils Affected by Apple Replant Disease

**DOI:** 10.3389/fmicb.2022.841558

**Published:** 2022-03-25

**Authors:** Felix Mahnkopp-Dirks, Viviane Radl, Susanne Kublik, Silvia Gschwendtner, Michael Schloter, Traud Winkelmann

**Affiliations:** ^1^Section Woody Plant and Propagation Physiology, Institute of Horticultural Production Systems, Leibniz Universität Hannover, Hanover, Germany; ^2^Research Unit for Comparative Microbiome Analysis, Helmholtz Zentrum München, Munich, Germany

**Keywords:** endophyte, apple replant disease (ARD), *Malus domestica*, *Streptomyces*, microbiome, *Pseudomonas*

## Abstract

Apple replant disease (ARD) is a worldwide problem for tree nurseries and orchards leading to reduced plant growth and fruit quality. The etiology of this complex phenomenon is poorly understood, but shifts of the bulk soil and rhizosphere microbiome seem to play an important role. Since roots are colonized by microbes from the rhizosphere, studies of the endophytic microbiome in relation to ARD are meaningful. In this study, culture-independent and culture-dependent approaches were used in order to unravel the endophytic root microbiome of apple plants 3, 7, and 12 months after planting in ARD-affected soil and ARD-unaffected control soil at two different field sites. Next to a high diversity of *Pseudomonas* in roots from all soils, molecular barcoding approaches revealed an increase in relative abundance of endophytic Actinobacteria over time in plants grown in ARD and control plots. Furthermore, several amplicon sequence variants (ASVs) linked to *Streptomyces*, which had been shown in a previous greenhouse ARD biotest to be negatively correlated to shoot length and fresh mass, were also detected in roots from both field sites. Especially in roots of apple plants from control soil, these *Streptomyces* ASVs increased in their relative abundance over time. The isolation of 150 bacterial strains in the culture-dependent approach revealed a high diversity of members of the genus *Pseudomonas*, confirming the data of the molecular barcoding approach. However, only partial overlaps were found between the two approaches, underlining the importance of combining these methods in order to better understand this complex disease and develop possible countermeasures. Overall, this study suggests a key role of *Streptomyces* in the etiology of ARD in the field.

## Introduction

Apple replant disease (ARD) is a worldwide complex problem, which affects apple tree nurseries and orchards, causing reductions in tree growth, fruit yield, and quality (Mazzola and Manici, 2012; Manici et al., 2013; Winkelmann et al., 2019). It occurs when apple is repeatedly planted at the same site and is defined as a “harmfully disturbed physiological and morphological reaction of apple plants to soils that faced alterations in their (micro-)biome due to previous apple cultures” (Winkelmann et al., 2019). The exact etiology of ARD is still not known, but there is increasing evidence that, next to changes in the abundance of specific pathogens, shifts of the bulk soil and rhizosphere microbiome are an important driver of ARD (Winkelmann et al., 2019; Balbín-Suárez et al., 2021). With the rise of next-generation sequencing, several studies revealed shifts of the rhizosphere microbiome community structure (Sun et al., 2014; Franke-Whittle et al., 2015; Yim et al., 2015; Nicola et al., 2018; Tilston et al., 2018; Balbín-Suárez et al., 2021) and showed an enrichment of potential ARD fungal pathogens [*Acremonium*, *Fusarium*, *Cylindrocarpon* (Franke-Whittle et al., 2015), *Pythium* (Tilston et al., 2018), *Ilyonectria*, and *Nectria* sp. (Balbín-Suárez et al., 2021)] and several bacteria associated with ARD [*Lysobacter*, *Pseudomonas* (Sun et al., 2014), *Chitinophaga*, *Hyphomicrobium* (Franke-Whittle et al., 2015), *Streptomyces*, and *Variovorax* (Balbín-Suárez et al., 2021)].

The plant endobiome is strongly influenced by colonization from the rhizosphere microbiome and, thus, is very likely subjected to changes in ARD-affected soil. Moreover, beneficial endophytes can support plants in coping with abiotic and biotic stresses. Therefore, analyses of the root endobiome of apple in replant-affected soils is of interest to understand the disease etiology, but also to develop mitigation strategies. However, studies of the endophytic microbiome and its role in ARD are rare and were mostly focused on fungal pathogens in apple roots. Kelderer et al. (2012) identified *Cylindrocarpon* spp. and *Rhizoctonia* sp. as pathogenic root endophytes in row (ARD affected) and inter-row (control) planted apple trees. *Fusarium oxysporum* and *Fusarium solani* were most abundant in roots in this study, but not considered as pathogens. Root endophytic *Cylindrocarpon*-like fungi (*Thelonectria* sp. and *Ilyonectria* spp.) were also shown by Manici et al. (2013) next to *Pythium* spp. to be correlated to the growth reduction in the rootstock M9 growing in ARD-affected soil. Different species of Nectriaceae were also found in ARD-affected cortex cells applying laser microdissection (Popp et al., 2020). Several fungal endophytes from ARD-affected apple roots were isolated and re-inoculated in a soil free biotest by Popp et al. (2019). Negative effects on plant health were reported for *Cadophora*, *Calonectria*, *Dactylonectria*, *Ilyonectria*, and *Leptosphaeria*.

For bacterial root endophytes, even less data are available. Only two studies were conducted, which investigated the role of bacterial endophytes to the context of ARD (Tewoldemedhin et al., 2011; Van Horn et al., 2021). Tewoldemedhin et al. (2011) used a cultivation-dependent approach to investigate the biocontrol properties of Actinobacteria isolates (92 isolates belonging to the genus *Streptomyces* and four to *Nocardiopsis*) from ARD-affected roots but inoculation of selected isolates resulted in no effects on plant growth. Amplicon sequencing was used by Van Horn et al. (2021) to characterize the endophytic community structure of rootstock genotypes reported to be susceptible (M26 and M9) and tolerant (G210, G41, G890, and G935) to ARD. The strongest community differences were found between tolerant and susceptible genotypes and the overall most abundant endophytes were members belonging to the genera *Arthrobacter*, *Burkholderia*, *Halospirulina*, and *Streptomyces*. In a previous study (Mahnkopp-Dirks et al., 2021), we conducted a biotest with apple plants of the ARD-sensitive rootstock genotype M26 grown in three soils differing in soil properties and compared for each soil the bacterial root endophytic community of plantlets grown in ARD-affected soil or control (grass) soil using a molecular barcoding approach. Results showed several amplicon sequence variants (ASVs) linked to *Streptomyces*, which were exclusively found in plants grown in ARD soil. Moreover, these ASVs were negatively correlated to shoot length and shoot fresh mass. These results were achieved for young plantlets (8 weeks of age) under controlled greenhouse conditions using *in vitro* propagated plant material, which did not represent field conditions. To validate the relevance of the observations of our greenhouse biotest under field conditions and to assess seasonal dynamics, we performed a field experiment at the two sites, from which the soils for the biotests were obtained. The bacterial root endophytic community structure in plants grown in ARD-affected or grass control soil using seed-propagated rootstocks (cv. ‘Bittenfelder Saemling’, hereafter referred to as Bittenfelder) was characterized. Samples were taken over 1 year to cover a complete vegetation cycle. We postulated that overall diversity and seasonal dynamics of bacterial root endophytes are less pronounced in plantlets grown in ARD-affected soils, as in these soils single bacterial endophytes dominate the microbiome of root endophytes, which negatively impact plant growth and overall plant performance. Moreover, we intended to obtain isolates to complement the culture-independent data and to serve as potential inoculants. Thus, we also used a culture-dependent approach in order to isolate a broad spectrum of bacterial root endophytes, established pure cultures, and identified them based on their 16S rRNA gene sequence. This will enable us to study their effects on apple plants and potentially to help and overcome the complex ARD phenomenon.

## Materials and Methods

### Field Sites and Sampling

The field experiments were carried out at two different sites in northern Germany: Heidgraben (x-coordinate 53.699199; y-coordinate 9.683171; WGS 84, Schleswig-Holstein) and Ellerhoop (x-coordinate 53.71435; y-coordinate 9.770143 WGS 84, Schleswig-Holstein), which differed in their soil properties (Mahnkopp et al., 2018). Based on World Reference Base for soil resources, the textures of the top soil (0–20 cm) of the two sites were classified as sand (Heidgraben) and loamy sand (Ellerhoop) (Mahnkopp et al., 2018). In 2017, the annual mean temperature for both sides was 10°C, and the annual precipitation reached 1,142 mm.

At both sites, two different variants were established, each in four replicates (plots): (i) ARD plots, where ARD was successfully induced by repeatedly replanting Bittenfelder apple seedling rootstocks since 2009 in a 2-year cycle, and (ii) control plots, which were covered with grass since then. In spring 2016, at Heidgraben and spring 2017 at Ellerhoop, one-third of these grass control plots were planted with Bittenfelder plants representing the first apple planting generation (hereafter referred to as grass plots). ARD plots at Ellerhoop were replanted for the last time in spring 2017 and at Heidgraben in spring 2016 representing the fifth replant generation at the time of sampling (Table 1). Both sites were managed as closely as possible to nursery practice and were treated in a similar way. However, since the apple plants were grown not only at the two different sites but also in two different years, the management was not completely the same. Per year, each site was fertilized with 54 kg of nitrogen per hectare.

**TABLE 1:** Experimental setting and sampling time points at the two sites “Heidgraben” and “Ellerhoop” (1 = sampling in July, 2 = sampling in November, and 3 = sampling in April); *n* = 4 plots with 3 plants each.

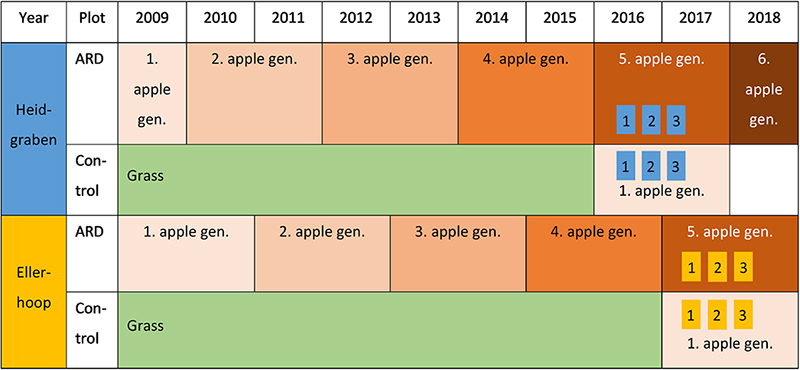

The planting material for this study was obtained from a specialized nursery (Stahl Baumschulen GmbH, Tornesch, Germany) as 1-year-old Bittenfelder seedlings. These were sown in spring 2015 and 2016, respectively, on a loamy sandy soil (fertilized with 95 kg nitrogen per hectare), uprooted in the following autumn, and stored over winter in a cooling chamber. At Heidgraben, plants were planted on April 5 and 6, 2016 for both plots; at Ellerhoop, April 10 and 11, 2017. At both sites, plants were sampled at three time points after planting: 3 months after planting (summer), 7 months after planting (late autumn), and 1 year after planting (spring). At Heidgraben, sampling was performed on July 27, 2016 (summer), November 16, 2016 (autumn), and April 25, 2017 (spring); at Ellerhoop, on July 25, 2017 (summer), November 13, 2017 (autumn), and April 23, 2018 (spring). Three plants were sampled per plot and sampling time point (in total 24 per site and season).

To determine the root fresh weight of plants taken after 12 months (spring), adhering soil was removed by carefully washing the roots under running tap water until no soil was visible any more followed by briefly drying the roots using paper towels. Since fresh roots were analyzed further by project partners, determining the root dry weight was not possible. However, in previous studies with apple roots (Mahnkopp et al., 2018), we observed a highly significant Pearson correlation [*r*(214) = 0.97, *p* < 0.05] between root fresh mass and root dry mass. In addition, samples of the planting material (plants taken before the transfer into the soil) were taken at Ellerhoop in spring 2017 and served as “time point zero” (T0) plants.

### Root Surface Disinfection

The following surface disinfection was performed as described in Mahnkopp-Dirks et al. (2021): To get rid of the adhering soil, roots were washed carefully. Afterward, they were rinsed for 30 s in EtOH (70%), followed by stirring in 2% NaOCl for 7.5 min and finally washing five times in sterile deionized water. The final washing water was plated on 523 medium (Viss et al., 1991) and R2A Agar (Reasoner and Geldreich, 1985) and incubated for 1 week at room temperature. Plating resulted in <10 CFU per plate in all cases. Roots were stored in sterile 2-ml Eppendorf tubes at −80°C until DNA extraction for amplicon sequencing.

### Molecular Barcoding of Root Endophytic Bacteria

For Illumina sequencing, DNA was extracted as mentioned in Mahnkopp-Dirks et al. (2021) using the Invisorb Spin Plant Mini Kit (Stratec, Berlin, Germany) according to the manufacturer’s instructions. The extracted DNA of three biological replicates was pooled. For each sampling time point per site, four replicates (pooled DNA) were used for sequencing. For seven samples, no PCR product could be amplified, resulting in reduced replicate number (Table 2).

**TABLE 2:** Richness and diversity of endophytic bacterial communities based on amplicon sequence variants (ASVs) in roots from Bittenfelder plants grown in apple replant disease (ARD) plots or grass plots at the sites Heidgraben and Ellerhoop.

Site	Soil	*n*	Observed ASVs	Chao1	Shannon	Simpson
Ellerhoop	T0	3	244 ± 26	253 ± 30	4.6 ± 0.12	0.98 ± 0.00

Heidgraben (Summer 16)	ARD	3	260 ± 39	266 ± 41	5.0 ± 0.19	**0.99 ± 0.00**
	
	Grass	4	210 ± 78	222 ± 85	4.19 ± 0.47	0.96 ± 0.02*

Heidgraben (Autumn 16)	ARD	4	291 ± 96	301 ± 104	4.82 ± 0.43	0.98 ± 0.01
	
	Grass	4	240 ± 95	248 ± 100	4.48 ± 0.59	0.97 ± 0.02

Heidgraben (Spring 17)	ARD	4	251 ± 41	264 ± 39	4.85 ± 0.31	0.99 ± 0.01
	
	Grass	4	236 ± 21	243 ± 19	4.76 ± 0.24	0.98 ± 0.01

Ellerhoop (Summer 17)	ARD	4	236 ± 72	245 ± 77	4.42 ± 0.61	0.97 ± 0.02
	
	Grass	3	166 ± 43	175 ± 43	3.89 ± 0.49	0.96 ± 0.02

Ellerhoop (Autumn 17)	ARD	2	160 ± 21	162 ± 22	4.05 ± 0.01^a^	0.96 ± 0.01
	
	Grass	3	171 ± 41	176 ± 37	4.04 ± 0.96	0.94 ± 0.06

Ellerhoop (Spring 18)	ARD	3	253 ± 77	263 ± 83	4.72 ± 0.17^b^	0.98 ± 0.00
	
	Grass	4	159 ± 28	161 ± 29	4.06 ± 0.73	0.92 ± 0.10

Additionally, T0 plants before planting at Ellerhoop are shown. Different letters indicate significant differences within the sites between the sampling times (Tukey’s test at p ≤ 0.05). No letters indicate no significant differences. Significant differences between ARD and grass are shown in bold (t-test at *p ≤ 0.05). Given are mean ± standard deviation of n replicates.

Amplicon sequencing was done using the primer combination 335F (CADACTCCTACGGGAGGC)/769R (ATCCTGTTTGMTMCCCVCRC) (Dorn-In et al., 2015) to amplify the V3–V4 region of the 16S rRNA gene. Amplicon library preparation and bioinformatics analysis were described in detail in Mahnkopp-Dirks et al. (2021). Briefly, PCR was performed using 2x Phusion High-Fidelity Master Mix (Thermo Fisher Scientific, Waltham, MA, United States), 10 pmol of each primer, and 5 ng of DNA template in a final volume of 10 μl with PCR conditions: 98°C for 10 s, 30 cycles of 98°C for 1 s–59°C for 5 s–72°C for 45 s, 72°C for 1 min. After purification with Agencourt AMPure XP kit (Beckman Coulter, United States) indexing PCR (98°C for 30 s, 8 cycles of 98°C for 10 s–55°C for 30 s–72°C for 30 s, 72°C for 10 min) was performed using Nextera XT Index Kit v2 (Illumina, United States). Purified samples were equimolarly pooled to 4 nM and sequenced on Illumina Miseq platform. FASTQ files were trimmed using AdapterRemoval (Schubert et al., 2016) and analyzed using the QIIME 2 software package release 2017.11 (Caporaso et al., 2010) with default parameters. Quality control was performed *via* QIIME 2 plugin DADA2 (Callahan et al., 2016), with removal of 10 bp n-terminally, length truncation at position 300 (forward) and 260 (reverse), and an expected error of 2. Taxonomic assignment of the resulting ASVs was performed using primer-specific pre-trained Naive Bayes classifiers of the SILVA_132_QIIME release 99% and the q2-feature-classifier plugin. Raw sequence data were deposited in GenBank^1^ under the BioProject accession number PRJNA795995.

PCR-negative control showed no ASVs; thus, contamination during sample processing could be excluded. For further data analysis, unassigned reads, singletons, plastid sequences, and sequences assigned to archaea and eukaryotes were removed (in sum 37% of all reads), resulting in total in 4,694 different ASVs, of which 4,422 (94.2%) were covered after rarefying at 4,213 reads (Supplementary Figure 2). The relative abundance was calculated by dividing the number of reads per ASV in the samples by the sum of total reads per sample and finally multiplied by 100. To calculate the overall relative abundance of the corresponding phylum/genus, ASVs belonging to the same phylum/genus were merged.

Determination of species diversity (Shannon and Simpson) and richness (Chao1) indices of the amplicon data was done using the “Phyloseq” (McMurdie and Holmes, 2013) and “Vegan” (Oksanen et al., 2019) packages of R v3.6.1 (R Development Core Team, 2019).^2^ Normal distribution based on Shapiro-Wilk test (Shapiro and Wilk, 1965) and homogeneity of variance based on Levene’s test (Levene et al., 1960) were tested using the program PAST3 v. 3.20 (Hammer et al., 2001). If the null hypotheses of normal distribution and equal variances were rejected, the Tukey test based on Herberich et al. (2010) was used at *p* < 0.05 to determine significant differences of the diversity and richness scores. In order to compare the relative abundance of different phyla in different seasons and ARD variants to grass variants, a DESeq2 analysis using generalized linear models and pairwise comparisons (*p* < 0.05) was performed [DESeq2, Love et al. (2014)]. Non-metric multidimensional scaling (NMDS) was performed with the program PAST3 v. 3.20 (Hammer et al., 2001) using the Bray–Curtis similarity index and analysis of similarity (ANOSIM) in order to visualize the community composition of the different samples. Vectors showing the correlation between the corresponding genus and the NMDS score were added to indicate the influence of the corresponding genera.

### Isolation of Bacterial Root Endophytes

In order to isolate bacterial endophytes, four random 1-cm pieces of surface disinfected fine roots (Ø < 2 mm) of each plant were placed per Petri dish containing 523 (Viss et al., 1991) and R2A medium (Reasoner and Geldreich, 1985). For each plant, three Petri dishes per medium were prepared as replicates. After approximately 7 days at room temperature, colonies were picked based on different morphology and purified by dilution plating using the same media.

To avoid slow-growing colonies to be overgrown by fast-growing bacteria, additionally 100 mg of surface-disinfected roots were cut into small pieces and transferred into a 50-ml centrifuge tube containing 10 ml of saline (0.85% NaCl). Samples were shaken at 150 rpm and 4°C for 22 h. One hundred microliters of the solution as well as dilutions up to 1:10^5^ were plated onto three Petri dishes containing 523 medium and R2A medium, respectively, and evenly distributed. After 7–28 days, colonies were picked and streaked out. Selection of different colonies was based on different appearance and morphology with the aim to obtain a broad spectrum of different bacterial root endophytes.

Single colonies were transferred to liquid medium 523 and incubated for 1–7 days at room temperature on a shaker at 150 rpm until growth was visible. One milliliter of this suspension was used for DNA extraction based on the protocol of Quambusch et al. (2014).

Partial sequences of the 16S rRNA gene of 140 isolates were obtained using the primers 27f (AGAGTTTGATCCT GGCTCAG) and 1492r (GGYTACCTTGTTACGACTT) (Weisburg et al., 1991). Each PCR reaction (25 μl) contained 10 ng of DNA, 1 × Williams Buffer (100 mM Tris–HCl, pH 8.3 at 25°C; 500 mM KCl; 20 mM MgCl_2_; 0.01% gelatin), 200 μM dNTPs, 10 pmol of each primer, and 1 U Biotaq DNA polymerase (Bioline, London, United Kingdom). The thermal cycler protocol started with an initial denaturation at 94°C for 5 min, followed by 35 cycles of denaturation at 94°C for 30 s, annealing of the primers at 52°C for 40 s, and elongation at 72°C for 60 s, and ended with a final elongation at 72°C for 5 min.

Fragments were separated *via* gel electrophoresis [1 × Tris–acetate-EDTA (TAE) buffer, Aaij and Borst, 1972; Hayward and Smith, 1972] and the PCR products of about 1,500 bp were excised from 1% agarose gels and purified using the NucleoSpin Gel and PCR Clean-up Kit (Macherey and Nagel, Düren, Germany). The 16S rRNA gene fragments were sequenced with the Sanger method (Sanger et al., 1977) by Microsynth Seqlab (Göttingen, Germany) using the same primers as described above.

For phylogenetic analysis, an alignment of the nucleotide sequences of 150 isolates was done using BioEdit (version 7.2.5, Hall, 1999). Therefore, all sequences were cut at 1,320 bp before ClustalW multiple alignment (Thompson et al., 1994) was done with the number of bootstraps set to 1,000, resulting in a total of 62 different sequences that were deposited in GenBank (see footnote text 1) under the accession MW580614:MW580673[accn]. This alignment was used for phylogenetic tree construction with the program MEGA X (Kumar et al., 2018) using the Maximum Likelihood method and Tamura–Nei model (Tamura and Nei, 1993). Identities and origins of the different isolates can be seen in Supplementary Table 1.

To link the culture-independent approach and the culture-dependent approach, a local Blastn of the 16S rRNA sequences of the isolates against all ASVs obtained from amplicon sequencing was done using BioEdit (version 7.2.5, Hall, 1999).

## Results

After 12 months of growing in ARD or grass plots, roots of apple plantlets showed clear differences (Table 3 and Supplementary Figure 1). Roots of plants from Ellerhoop growing in ARD soil had significantly lower mass (21.64 ± 10.91 g) compared to roots from those in the grass soil (78.21 ± 33.49 g). At Heidgraben, roots from ARD soil had a lower mass (36.88 ± 15.65) than roots grown in grass soil (52.61 ± 25.34 g), but the differences were not significant due to high plant-to-plant variation.

**TABLE 3:** Fresh weight of roots after 12 months of growth in ARD or grass plots at Heidgraben and Ellerhoop.

	ARD	Grass
Heidgraben	36.88 ± 15.65	52.61 ± 25.34
Ellerhoop	21.64 ± 10.91^a^	78.21 ± 33.49^b^

*Different letters indicate significant differences between ARD and grass variants within a site (Welch Two Sample t-test p ≤ 0.05, n = 12).*

### Molecular Barcoding of Bacterial Root Endophytes

In order to compare the bacterial diversity of endophytes of roots growing in ARD-affected and non-affected soils, a metabarcoding approach using extracted DNA from the roots after surface disinfection and 16S rRNA gene amplification was performed.

Overall, the richness (observed ASVs, Chao1) of bacterial endophytes was higher in roots grown in soil at Heidgraben compared to Ellerhoop. Seasonal fluctuations at both sites differed: Whereas the highest numbers of observed ASVs were found in autumn (2017) at Heidgraben, at Ellerhoop, at the same time point of the experiment (autumn 2018), the numbers of observed ASVs at Ellerhoop were the lowest (Table 2). In five out of six variants (site + season), richness of bacterial ASVs in roots grown in ARD soil was higher compared to roots from plants grown in grass soil, but the differences did not reach the level of significance due to high variations between replicate samples. Evenness (Simpson index) of root endophytes was not affected by the sampling site or by season and treatment.

**FIGURE 1:**
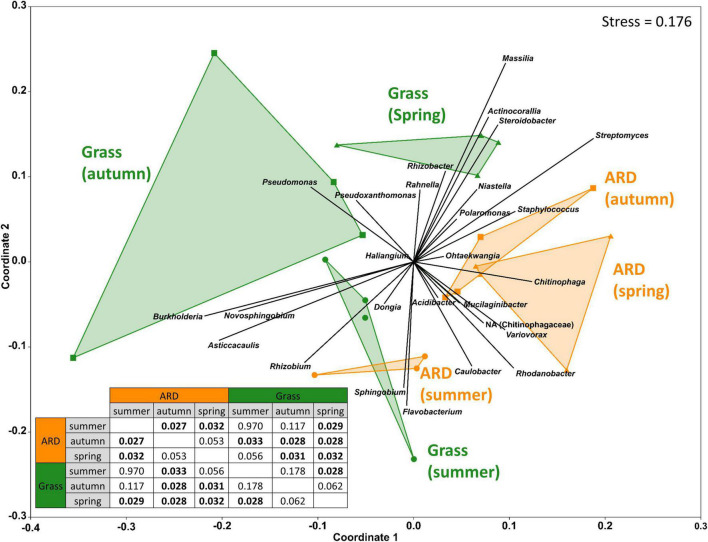
Three-dimensional non-metric multidimensional scaling (NMDS) using Bray–Curtis dissimilarity of roots taken in summer and autumn after planting or the following spring at Heidgraben. Vectors represent the correlation coefficient between the corresponding genus and the NMDS score. Relative lengths and the directions of the vectors indicate the influence of the respective genera (RA > 0.5%). The third axis is not shown. Results of the one-way analysis of similarities are shown in the lower left corner; significant differences are highlighted in bold (*p* ≤ 0.05).

The large plant-to-plant variation is also depicted in the results of β diversity analyses. At both sites, roots from grass plots clustered separately from those grown in ARD plots, except for the first sampling 3 months after planting (summer, Figure 1 and Supplementary Figure 3). For the separation of samples from grass and ARD plots, several bacterial genera had an effect, such as *Pseudomonas, Burkholderia, Chitinophaga*, or *Streptomyces* at Heidgraben (Figure 1) and *Pseudomonas*, *Halomonas*, *Streptomyces*, or *Sphingobium* at Ellerhoop (Supplementary Figure 3). For samples from Ellerhoop, endophytic bacterial communities of the planting material (T0) were clearly different from those of plants grown for 3–12 months in the different field plots (Supplementary Figure 3).

#### Proteobacteria

**FIGURE 2:**
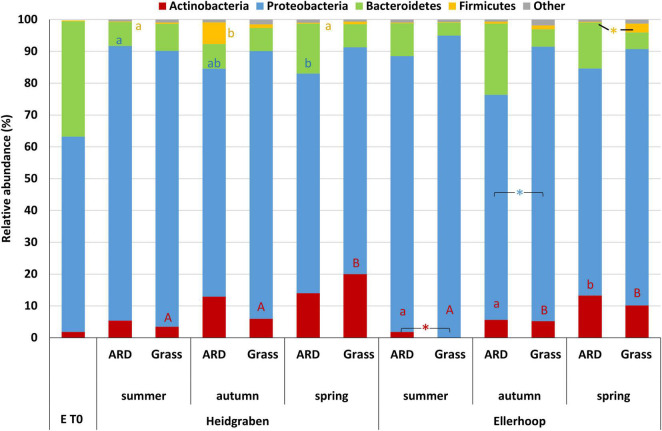
Relative abundance of dominant phyla in roots of Bittenfelder plants grown in apple replant disease (ARD) plots or grass plots at Heidgraben and Ellerhoop taken in summer and autumn after planting or the following spring. Different letters indicate statistically significant differences within one site in ARD plots (lower case) or grass plots (upper case) between the seasons (DESeq2 analysis using a generalized linear model and multiple comparisons with *p* ≤ 0.05). Significant differences between ARD and grass within one season are indicated by an asterisk (DESeq2 analysis using a generalized linear model and pairwise comparisons with *p* ≤ 0.05). Different colored letters belong to the respective phyla. *N* numbers are shown in Table 2.

Proteobacteria were the dominant bacterial phylum detected as root endophytes with a mean relative abundance of 77.3% in all samples (Figure 2). Significant differences in the relative abundance of Proteobacteria between roots from plants grown in ARD and control soil were only detected for Ellerhoop (autumn) with a reduced relative abundance in roots grown in ARD-affected soil. The most abundant proteobacterial genus was *Pseudomonas* (mean of relative abundance over all samples 20.1%).

**FIGURE 3:**
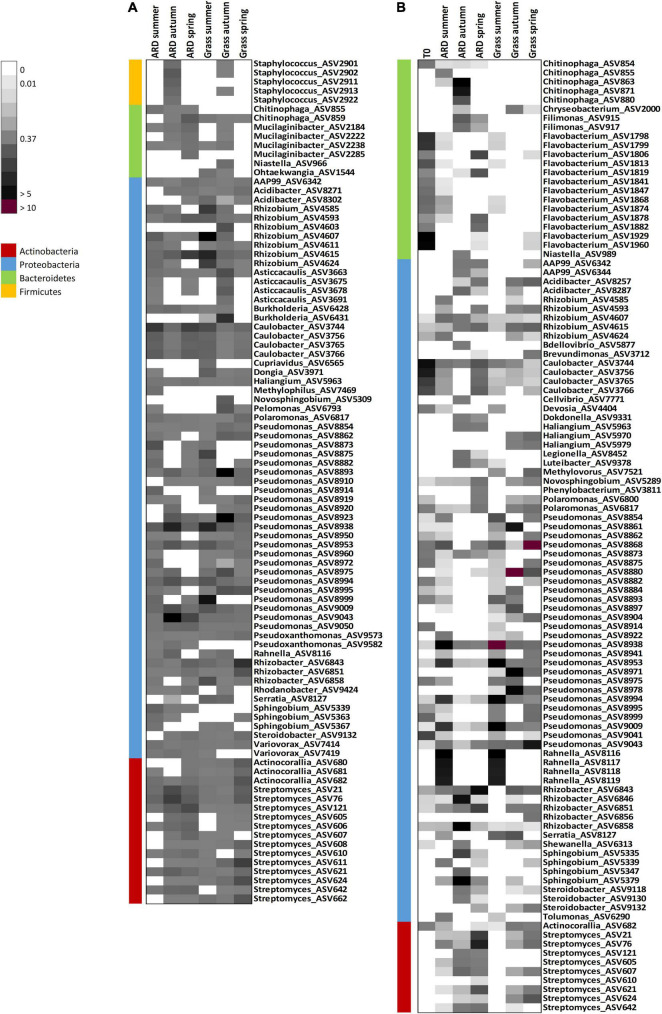
Heatmap showing the abundance of different amplicon sequence variants (ASVs) (RA > 0.5%) in roots of Bittenfelder plants from ARD and grass plots taken 3 months (summer), 7 months (autumn), and 12 months (spring) after planting at Heidgraben **(A)** in 2016/17 and Ellerhoop **(B)** in 2017/18. For each sampling time per site and soil, only ASVs with an abundance greater than 0.5% were selected, and their relative abundance was compared with all other variants. The color code indicates the range from low relative abundance (light gray, 0.01%), medium abundance [gray, 0.37% (median)], to high abundance (black > 5%, purple > 10%). Different colors on the left side indicate the corresponding phylum of the ASVs.

At Ellerhoop, *Pseudomonas* showed a different development in roots grown in grass soil compared to ARD soil over time. In grass soil, *Pseudomonas* was constantly the dominating genus at all sampling times (sum of RA of all *Pseudomonas* ASVs in summer = 35.6%, autumn = 34%, spring = 31.1%), whereas in ARD soil, the relative abundance decreased over time (sum of RA in summer = 20%, autumn = 1.4%, spring = 2.3%; Figure 3).

At Heidgraben, the overall relative abundance of *Pseudomonas* showed a similar pattern between roots grown in ARD and grass soil. In both soils, roots had the highest relative abundance of *Pseudomonas* in autumn (ARD = 20%, Grass = 23.5%) and decreased over time to the lowest abundance in spring (ARD = 8.6%, Grass = 11%).

Amplicon sequence variants linked to *Pseudomonas* were not only most abundant in the analyzed root samples, but also highly diverse. Overall, 34 ASVs could be detected (based on RA > 0.5), with a slightly higher number of differing ASVs obtained from Ellerhoop compared to Heidgraben (26 and 23 for Ellerhoop and Heidgraben, respectively). Most of these ASVs were classified as *P. corrugata* and *P. turukhanskensis* (14.7% each). While the number of ASVs linked to *Pseudomonas* at both sites in roots grown in grass soil slightly decreased after summer (Heidgraben summer = 22, autumn = 18, spring = 19; Ellerhoop summer = 26, autumn = 16, spring = 18), the number continuously decreased even more in roots grown in ARD sites at Heidgraben (summer = 20, autumn = 16, spring = 14) and especially after summer at Ellerhoop (Ellerhoop summer = 22, autumn = 6, spring = 8).

Twenty-seven ASVs linked to *Pseudomonas* were present in roots from T0 plants. Most of them disappeared over time in roots grown in ARD soil. After 1 year (spring), only 7 out of 27 ASVs were still present in roots grown in ARD soil. However, in roots grown in grass soil, 16 ASVs were still detected, most of them being highly abundant.

Interestingly, 15 ASVs related to *Pseudomonas* were present at both sites at Ellerhoop and Heidgraben. Whereas these ASVs showed different development at ARD plots at both sites in response to sampling time and treatment, 7 ASVs linked to *Pseudomonas* showed a similar development at grass plots and were mainly decreasing in abundance over time (Figure 3, ASV8873, ASV8914, ASV8938, ASV8953, ASV8994, and ASV9009).

The second most abundant proteobacterial genus was *Rhizobium* (mean of relative abundance over all samples 4.5%). While, in both sites, the overall relative abundance of *Rhizobium* showed the same pattern over time in roots grown in ARD soil (Heidgraben sum of RA in summer = 4.3%, autumn = 2.7%, spring = 4.3%; Ellerhoop in summer = 2.9%, autumn = 1.4%, spring = 2%), a different pattern was observed in roots grown in grass soil. At Heidgraben, the overall relative abundance decreased (sum of RA in summer = 15.3%, autumn = 5.7%, spring = 1%), while it increased at Ellerhoop over time (sum of RA in summer = 0.3%, autumn = 1.9%, spring = 2.3%). In total, seven different ASVs linked to *Rhizobium* were found in roots from Heidgraben and 5 ASVs were found in roots from Ellerhoop (which were all shared between both sites). From a total of 7 ASVs, three were classified as *R. etli* and three as *R. alamii*. No clear patterns in number of ASVs over time were observed.

Amplicon sequence variants linked to the proteobacterial genus *Rhanella* were only found at Ellerhoop in autumn but with high abundance in roots grown in both soils (ARD = 20%; grass = 19.9%).

#### Bacteroidetes

The second most abundant phylum was Bacteroidetes (average abundance 12.8% across all samples). Here, no significant differences in overall relative abundance between plants grown in control (grass) soils and ARD-affected soils were detected. The most abundant genera in the group of Bacteroidetes were *Flavobacterium* (2.99%), *Mucilaginibacter* (1.3%), and *Chitinophaga* (1.16%, average over all samples). However, *Flavobacterium* was only present at Ellerhoop. Interestingly, 13 ASV linked to *Flavobacterium* were present in T0 plants, which was clearly the dominating genus with 23.9%. However, the number of ASVs linked to *Flavobacterium* decreased after planting in ARD soil (summer = 5, autumn = 0, spring = 1), and after initially decreasing over time, it slightly increased again in grass soil (summer = 8, autumn = 1, spring = 6).

In total, 7 ASVs linked to *Chitinophaga* were found (5 at Ellerhoop and 2 at Heidgraben). Only 1 ASV was found in both sites and classified as *C. ginsengisoli*. Except for ASV859 (classified as *C. oryziterraeno*), ASVs linked to *Chitinophaga* were not present in roots grown in grass soil. At Ellerhoop, the overall relative abundance of C*hitinophaga* peaked in autumn (sum of RA in summer = 0.66%, autumn = 12.01%, spring = 0.02%).

#### Actinobacteria

Actinobacteria had a mean relative abundance of 7.5% in average, with higher abundance observed for Heidgraben (Figure 2). Interestingly, the relative abundance for this phylum increased over time in ARD and grass plots at both sites. Significant differences between ARD-affected and grass plots were observed for Actinobacteria when root samples from Ellerhoop (summer) were compared, with higher numbers in roots from plants obtained from ARD-affected plots.

The most abundant actinobacterial genus was *Streptomyces*. Their relative abundance increased over time in roots grown in ARD-affected soil at Heidgraben (sum of RA in summer = 3.36%, autumn = 10.93%, spring = 10.38%) and Ellerhoop (sum of RA in summer = 0.84%, autumn = 3.81%, spring = 11.65%), making *Streptomyces* the most abundant genus in these variants. An increase in relative abundance over time was also observed in roots grown in grass soil at Heidgraben (sum of RA in summer = 1.93%, autumn = 2.78%, spring = 11.65%) and Ellerhoop (sum of RA in summer = 0.00%, autumn = 1.20%, spring = 4.65%). A comparable pattern could be found for the diversity. In total, 13 different ASVs linked to *Streptomyces* were observed from which nine were shared between the sites. At both sites, the number of ASVs increased over time in roots grown in ARD-affected soil (Heidgraben: summer = 7, autumn = 13, spring = 13; Ellerhoop: summer = 6, autumn = 8, spring = 9) as well as in grass soil (Heidgraben: summer = 10, autumn = 12, spring = 12; Ellerhoop: summer = 0, autumn = 6, spring = 5). At both sites, most ASVs linked to *Streptomyces* increased in their relative abundance over time in roots grown in ARD-affected soil but especially in grass soil. Three out of 13 ASVs were classified as *S. camponoti*. No ASVs linked to *Streptomyces* were found in T0 plants.

#### Firmicutes

Firmicutes was a phylum with a low relative abundance (1.3%) and only found in roots from Heidgraben. This phylum was represented by five different ASVs linked to the genus *Staphylococcus*. In roots grown in ARD soil and grass soil, these ASVs were only present in autumn (ARD = 5 ASVs, Grass = 3 ASVs). Their relative abundance was higher in ARD (sum of RA = 4.9%) compared to grass soil (0.11%).

### Culture-Dependent Approach

**FIGURE 4:**
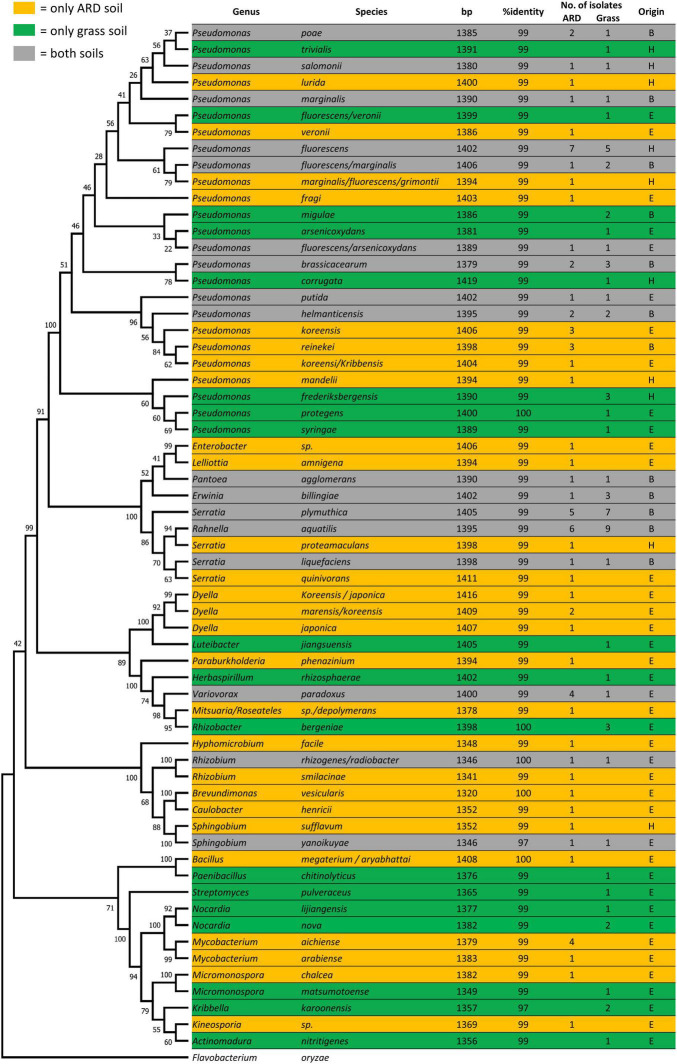
Phylogenetic tree based on 16S rRNA gene sequences of all different endophytic isolates using the Maximum Likelihood method and Tamura–Nei model. The percentage of replicate trees in which the associated taxa clustered together in the bootstrap test [1,000 replicates (Felsenstein, 1985)] are shown next to the branches. The closest hit with species level and corresponding identity using the NCBI database is shown. Only isolates with more than 1,300 bp were selected for alignment. *Flavobacterium oryzae* was used as an outgroup to root the tree. Isolates were obtained from roots grown only in ARD plots (yellow), grass plots (green), or both plots (gray) from the sites Heidgraben (H), Ellerhoop (E), or both (B) sites.

Next to molecular barcoding, a culture-dependent approach was performed in order to obtain a wide range of different endophytic bacterial isolates. In total, 150 isolates were obtained from both sites and sampling times (Figure 4), belonging to 69 different bacterial species and 29 genera. Thirty-one species were only found in roots grown in ARD soil, 19 only in grass soil, and 19 in both soils. Most species (25 out of 69) were classified as *Pseudomonas*, confirming the molecular data; 62.2% of all isolates obtained from Heidgraben and 31.9% from Ellerhoop were classified as *Pseudomonas*. At both sites, their distribution showed only slight differences between the percentage of isolates obtained from ARD or grass plots (Heidgraben: ARD = 59.1%, grass = 65.2%; Ellerhoop: ARD = 33.3%, grass = 30.2%).

Only one isolate classified as *Streptomyces* was found. Species that were isolated most frequently were *Rhanella aquatilis* (15), *Pseudomonas fluorescens* (12), and *Serratia plymuthica* (12).

To link the isolates obtained from the culture-dependent approach to the ASVs of the culture-independent approach, their 16S rRNA gene sequences were blasted against the sequences obtained from amplicon sequencing using a local Blastn. Nearly all isolates showed a very high similarity to one or more of the ASVs (Supplementary Table 2). However, only 20 Isolates out of 62 (>1,300 bp) showed a 100% identity to ASVs. The isolate *Kribella karoonensis* showed with 89.3% [to NA_ASV568 (Nocardioidaceae)] the lowest identity to the amplicon data followed by *Actinomadura nitritigenes* with 93.2% to NA_ASV677 (Non-omuraea). The isolates that were obtained frequently, e.g., *Pseudomonas fluorescens* (12x) or *Rhanella aquatilis* (15x), were only found in low abundance in the amplicon data. The five most abundant ASVs linked to *Pseudomonas* were classified as *P. brassicacearum* (ASV8938 and ASV9009), *P. corrugata* (ASV8893 and ASV8880), and *P. frederiksbergensis* (ASV9043), which were also identified in the culture-dependent approach (Supplementary Table 2).

## Discussion

### Community Structure and Relative Abundance Over Time

In most studies, in which rhizosphere or bulk soils of ARD-affected sites were analyzed (Sun et al., 2014; Franke-Whittle et al., 2015; Yim et al., 2015; Peruzzi et al., 2017; Tilston et al., 2018), Proteobacteria were the dominant phylum with a mean relative abundance of 35% (Nicola et al., 2018). However, a clearly higher relative abundance of Proteobacteria was observed, when the root endobiome was investigated in greenhouse-grown plants in the frame of an ARD biotest (Mahnkopp-Dirks et al., 2021) as root endophytes. These results were confirmed in the present study for roots grown in the field at Heidgraben and Ellerhoop. At both sites, Proteobacteria showed in roots grown in ARD plots an average relative abundance of 76%, respectively (Figure 2). However, in roots grown in grass soil, the relative abundance was even higher with an average of 84%. Due to their high metabolic activity and fast growth, members of this phylum are known to usually dominate the endosphere (Lundberg et al., 2012; Reinhold-Hurek et al., 2015).

During the first year of growth after planting, the community structure changed over time. This was also observed by Rumberger et al. (2007) for the bacterial rhizosphere community of apple trees grown in ARD-affected sites using terminal restriction fragment length polymorphism (T-RFLP) analyses. In the present study, the relative abundances of Actinobacteria increased over time in roots grown in ARD and grass plots at both sites (Figure 2). At the Heidgraben site, 12 months after planting (spring), the relative abundance was even higher in roots grown in grass plots than in ARD plots. Microscopic analysis revealed Actinobacteria to be more often found in roots grown in ARD-affected soil than in unaffected soil (Grunewaldt-Stöcker et al., 2019). Also, in-depth investigations of typical symptomatic root segments revealed a high frequency of Actinobacteria on the root surface and in the cortex (Grunewaldt-Stöcker et al., 2021). Actinobacteria were also observed in higher abundance in the previous greenhouse biotest in roots grown in untreated ARD soil in comparison to controls (Mahnkopp-Dirks et al., 2021). An increase of Actinomycetes (Actinobacteria) in the rhizosphere was also observed by Čatská et al. (1982) with increasing age of apple trees grown in ARD-affected soils.

Čatská et al. (1982) also reported a decline in “fluorescent pseudomonads” in apple trees within 30 months after planting in ARD-affected soil but not in control soil. A reduction of *Pseudomonas* in the rhizosphere over years after replanting confirmed by others (Rumberger et al., 2007; Jiang et al., 2017) was also observed for the endophytic root microbiome in the present study, especially at Ellerhoop in roots grown in ARD soil. Here, the total abundance of *Pseudomonas* ASVs was reduced 10-fold after summer but stayed nearly on the same level in roots grown in grass soil (Figure 3). This also points to the link between the rhizosphere and endosphere community since the main way of entering the root interior is through natural cracks during lateral root emergence and root tips (Hardoim et al., 2008; Bulgarelli et al., 2013). Members of *Pseudomonas* are known for their good rhizosphere competence and fast growth (Haas and Keel, 2003; Santoyo et al., 2012), and numerous strains are reported as plant growth promoting, e.g., by producing iron-chelating siderophores (Santoyo et al., 2012; David et al., 2018). These siderophores can prevent potential phytopathogens from acquiring (enough) soluble iron, thus inhibiting their growth and proliferation (Kloepper et al., 1980; Loper and Henkels, 1999; David et al., 2018). Furthermore, this trait of *Pseudomonas* was associated with disease-suppressive soils, among others (Kloepper et al., 1980). Moreover, several members, which are also associated with plants, are able to produce antibiotics (Rosales et al., 1995; Raaijmakers et al., 1997; Haas and Keel, 2003; Paulsen et al., 2005). Mazzola and Gu (2000) could show that a suppression of potential ARD causing pathogenic fungi was attributed to a transformation in composition of the fluorescent pseudomonad community in the apple rhizosphere with an increase in proportion of *Pseudomonas putida* in the population and a decrease in recovery of *P. syringae* and *P. fluorescens*. Therefore, it is possible that a decrease of the abundance of members of plant growth-promoting *Pseudomonas* could play a role in the establishment of ARD.

Overall, we could not verify a lower diversity of bacterial root endophytes in plantlets grown in ARD-affected soils. However, the dominance of single bacterial ASVs (e.g., from the group of *Streptomyces*) may lead to an outcompetition of other bacterial species with plant growth-promoting properties in the subsequent years, which may have a strong effect on plant performance.

Twenty months after planting, the apple rootstocks were uprooted, because both sites belong to a central experimental area on which ARD was induced and is now maintained by biannual replanting. Also in tree nurseries, rootstocks are cultivated only for one or two vegetation periods before being used for grafting. Thus, an observation of the bacterial endobiome over a longer time, which might have revealed even more pronounced changes, was not possible in this study.

### Apple Roots Grown in Field Soils Seem to Attract *Streptomyces*

The majority of Actinobacteria reads belonged to the genus *Streptomyces*, which had previously been suggested to play a role in ARD: In our greenhouse biotest (Mahnkopp-Dirks et al., 2021), we could show that the relative abundance of several unique ASVs linked to *Streptomyces* in roots from ARD soil from three different sites (including Heidgraben and Ellerhoop) was negatively correlated to shoot fresh mass and shoot length. Of these unique ASVs, 6 (Streptomyces_ASV76, 607, 611, 21, 121, and 621) and 5 (Streptomyces_ASV76, 607, 21, 621, and 121) were now detected in field-grown roots of the ARD plots at Heidgraben and Ellerhoop, respectively. Even though we observed a higher ARD severity [difference between fresh mass of roots growing in ARD and grass soil (Table 3)] in Ellerhoop, the overall relative abundance of *Streptomyces* was similar in both sites after 1 year (Heidgraben = 10.93%, Ellerhoop = 11.65%). However, two ASVs that were the most abundant in Ellerhoop (ASV21 and ASV76) were comparatively less abundant in Heidgraben, indicating the importance of the different ASVs. One of the most abundant unique *Streptomyces* ASVs in the greenhouse biotest, ASV76, which was present in 2016 at Heidgraben and Ellerhoop and 2017 at Heidgraben, was in total also the most abundant one in roots grown in ARD soil in the field sites Heidgraben and Ellerhoop. Overall, most ASVs linked to *Streptomyces* increased over time. Especially in roots grown in grass soil at Ellerhoop, where 3 months after planting in summer none of these *Streptomyces* ASVs were present, in the following spring, *Streptomyces* represented the second most abundant genus. With increasing root biomass over time, the total amount of root exudates is also increasing. It was shown that *Streptomyces* is more abundant in the rhizosphere of *Arabidopsis thaliana* (Badri et al., 2013; Lebeis et al., 2015) and their root colonization rate increased (Chewning et al., 2019) when plant exudates were present in comparison to when they were absent. Their accumulation could also lead to the assumption of pathogenicity of *Streptomyces*. After planting, their abundance is increasing over time. Even after removing the plant and planting non-Rosaceae for several years, *Streptomyces* could remain in high amount in the soil due to their ability to form spores, which can persist for years even under harsh conditions (Bobek et al., 2017). This would correlate with ARD, which is known to persist for decades after removing apple plants (Savory, 1966). After replanting apple, these highly abundant spores could germinate, triggered by plant material/exudates and therefore be a causative part of ARD. The question whether *Streptomyces* is phytopathogenic and could be a key player in ARD is discussed in detail in Mahnkopp-Dirks (2021) and Mahnkopp-Dirks et al. (2021). However, the accumulation of *Streptomyces* ASVs over time in roots grown in grass soil, which do not cause ARD symptoms, speaks against this hypothesis. However, *Streptomyces* is known to be able to suppress the plant defense response (Lehr et al., 2007; Tarkka et al., 2008; Vurukonda et al., 2018) by reducing the peroxidase activity and pathogenesis-related peroxidase gene (Spi2) expression and promote fungal root infections, which was shown in *Picea abies* (Lehr et al., 2007). This could mean that they enable easier colonization for potential fungal ARD pathogens. It was shown by Busnena et al. (2021) and Rohr et al. (2021) that some biphenyl and dibenzofuran compounds are present even under non-ARD conditions. Already at the first plant generation (here grass soil), *Streptomyces*, triggered and attracted by these phytoalexins, will accumulate over time. If apple is replanted, these plants face an already highly abundant *Streptomyces* population, which might reduce the plant defense response and enable easier colonization for potential fungal ARD pathogens.

In summary, in this study, we could show that the same *Streptomyces* ASVs identified previously in the biotest, which were negatively correlated to shoot length and shoot fresh mass, were also present in the field at Heidgraben and Ellerhoop during the season. Furthermore, in comparison to the biotest, Bittenfelder seedlings were used instead of the genotype M26. Thus, these ASVs linked to *Streptomyces* were associated with ARD independent of the genotype (Bittenfelder seedlings or M26), field or greenhouse, at two different sites and independent of seasons or years.

### Comparison of Culture-Dependent and -Independent Approach

Functional analyses of effects of certain endophytes often rely on inoculation experiments. Therefore, we conducted an additional culture-dependent isolation approach, which resulted in a collection of 150 bacterial isolates. To compare culture-dependent and culture-independent approaches, Liu et al. (2017) summarized the proportion of different endophytic bacterial phyla in different plants based on 25 different references. They found that root endophytic bacterial communities are typically dominated by Proteobacteria (≈50% in relative abundance), Actinobacteria (≈10%), Firmicutes (≈10%), and Bacteroidetes (≈10%). By 16S amplicon sequencing of xylem tissue from different apple genotype shoots, Liu et al. (2018) found the same four dominant different phyla, despite in slightly different relative abundance [Proteobacteria (58.4%), Firmicutes (23.8%), Actinobacteria (7.7%), and Bacteroidetes (2%)]. In the present study, the culture-independent 16S amplicon sequencing also revealed a root endophytic bacterial community dominated by Proteobacteria (80%), Bacteroidetes (9.7%), Actinobacteria (8.2%), and Firmicutes (1.2%) (Figure 2, mean of all plots and time points). The 150 isolates obtained in the present study by the culture-dependent approach were comparably dominated by Proteobacteria (85.3%), Actinobacteria (10%), and Firmicutes (2%). However, despite the so far similar phyla abundances between the culture-independent and -dependent approach, Bacteroidetes were not isolated.

In the culture-independent approach, 4,422 ASVs were found in total. They represent different sequences with at least 1 nucleotide difference, hence do not represent species level, which is often considered at a threshold of 97% sequence identity. Since the sequences of the 150 isolates all have at least one nucleotide difference, they would represent 3.4% of the total amount of ASVs found in the independent approach {sequencing errors cannot be excluded [Taq error rate ranges from 1.1 × 10^–4^ errors/bp (Barnes, 1992) to 8.9 × 10^–5^ errors/bp (Cariello et al., 1991)]}. The ASVs were linked to 473 different known genera. In the culture-dependent approach, isolates belonging to 29 different genera were obtained, which represent 6.13%. It is thought that only 0.1–10% of the total diversity of an environment is culturable (Handelsman and Smalla, 2003). Other studies indicate that more than 99% of all microorganisms are unculturable (Schloss and Handelsman, 2005; Vartoukian et al., 2010; Pham and Kim, 2012). Based on these numbers, the proportion of culturable bacteria in this study seems to be high. However, the total amount of 4,422 ASVs did not fully represent the total bacterial endophytic root community. Several biases in amplicon sequencing have an influence on the total bacterial endophytic root community (reviewed by Pollock et al., 2018). For instance, the universal primer pair used in our study for amplicon sequencing were chosen because of minimal non-target DNA amplification like mitochondrial or chloroplast DNA (Dorn-In et al., 2015). However, despite being “universal,” comparing the primer sequences to the 16S rRNA sequence collection of the Ribosomal Database Project (RDP, Cole et al., 2014) using “probe match” resulted in 1,122,475 hits out of 3,482,181 (32%) sequences in the domain Bacteria (when using 0 mismatches; 1 mismatch = 1,596,717; 2 mismatches = 1,910,059). Next to the primer used, the DNA extraction protocol has a strong influence on the bacterial community composition (Carrigg et al., 2007; Pollock et al., 2018).

Even though the two different culture media used resulted in several different cultured isolates, the number of potentially culturable bacterial endophytes will definitely increase with the use of more different media and physiological conditions. To also isolate obligate endophytes, the addition of plant extract to the medium might increase the number of different isolates (Eevers et al., 2015).

The most diverse genus in the culture-independent approach was *Pseudomonas*, with 138 ASVs linked to it. Likewise, isolates obtained from the culture-dependent approach belonging to the genus *Pseudomonas* were with 25 different species also the most diverse group. However, ASVs linked to the genus *Streptomyces* belonged to the most abundant ones, especially in roots grown in ARD soils, whereas in the culture-dependent approach, only one isolate could be obtained. One reason for this could be that the growth of *Streptomyces* was rather slow on the media used compared to other isolates, which might have outcompeted them. Another reason is that the outgrowth of isolates took place at room temperature. The optimal growth temperature for *Streptomyces* species is described as 28°C (Shepherd et al., 2010). Tewoldemedhin et al. (2011) were able to isolate 92 *Streptomyces* strains from surface-disinfected roots from six ARD-affected sites in South Africa using Casein-Starch medium and water agar supplemented with cycloheximide at 27°C for 4 weeks.

There were also some discrepancies in the abundance of some isolates compared to their corresponding ASVs (Supplementary Table 2). Several isolates were isolated frequently from roots, like *Rhanella aquatilis* or *Pseudomonas fluorescens*, but their corresponding ASVs were not found in high abundance in the amplicon sequencing. Reasons mentioned above like primer selection or DNA extraction methods could select against these bacteria in the culture-independent approach. Since both of these isolates were found to be fast growing on the media used, it is likely that the culture-dependent approach selected for them. Both genera were also isolated in high abundance from apple roots and rhizosphere soil by Dos Passos et al. (2014). With *Kribella karoonensis*, there was also one isolate, whose genus was not found in the culture-independent approach. The reason for this is probably that the primer 769R does not have any coverage in this genus based on 0 mismatches in the SILVA database. Several other isolates, like *Enterobacter*, *Lelliotti*, *Erwinia*, or *Rhanella*, were not found directly in the independent approach because the corresponding ASV sequences had several hits of different genera with the same score (resulting in NA), which means that the amplified sequence might not be long enough to discriminate between these genera. Discrepancies between culture-dependent and independent approaches were also observed in the phyllosphere of apple, where Actinomycetales were found only among isolates (Yashiro et al., 2011). In the present study, the culture-dependent approach was rather used as a qualitative method rather than a quantitative one to enable upcoming inoculation experiments.

## Conclusion

In this study, we provide evidence that the same six *Streptomyces* ASVs, which were found to be negatively correlated to shoot growth and fresh mass in a previous greenhouse biotest, were also found in high abundance in roots of a different rootstock cultivar grown in the field at two sites. Interestingly, most of these ASVs were increasing over time especially in newly planted apple plants in grass (virgin) soil leading to the assumption that the accumulation of these ASVs could play a role in ARD etiology. Furthermore, in this study, we could observe a decrease of the total abundance of *Pseudomonas* in the endophytic microbiome in roots grown in ARD soil, which may indicate that the presence of *Pseudomonas* is of importance for a balanced microbiome in healthy soils that is disturbed in ARD soils (dysbiosis). Next to the culture-independent approach, the isolation of 69 different bacterial strains showed on the one hand a comparable community structure with *Pseudomonas* being the most diverse genus. However, there is a need for further isolation efforts including different bacterial culture media and conditions in order to complement the collection of isolates, especially with regard to *Streptomyces*. On the other hand, the discrepancies between these two approaches underline the importance of combining different methods.

## Data Availability Statement

The datasets presented in this study can be found in online repositories. The names of the repository/repositories and accession number(s) can be found below: https://www.ncbi.nlm.nih.gov/genbank/, MW580614:MW580673[accn]; https://www.ncbi.nlm.nih.gov/genbank/, PRJNA795995.

## Author Contributions

TW conceived and designed the experiments. FM-D performed the experiments. FM-D, SG, SK, and VR analyzed the data. TW and MS contributed the reagents, materials, and analysis tools. FM-D, SG, SK, VR, TW, and MS contributed to writing the manuscript and approved the submitted version.

## Conflict of Interest

The authors declare that the research was conducted in the absence of any commercial or financial relationships that could be construed as a potential conflict of interest.

## Publisher’s Note

All claims expressed in this article are solely those of the authors and do not necessarily represent those of their affiliated organizations, or those of the publisher, the editors and the reviewers. Any product that may be evaluated in this article, or claim that may be made by its manufacturer, is not guaranteed or endorsed by the publisher.

## Footnotes

1
https://www.ncbi.nlm.nih.gov/genbank/

2
http://www.R-project.org

## Supplementary Material

Supplementary Figure 1 Apple roots in spring after 12 months of growth in grass soil (left) or ARD soil (right) at Ellerhoop (photo: Alicia Balbín-Suárez).

Supplementary Figure 2 Rarefaction curves showing the number of observed ASVs in all samples of Bittenfelder roots grown in ARD or grass soil at Heidgraben **(A)** and Ellerhoop **(B)** and taken in 3 months (summer), 7 months (autumn) or 12 months after planting (spring). Each line represents one sample of Bittenfelder roots taken in summer, autumn or spring. All samples were rarefied at 4,213 reads.

Supplementary Figure 3 Three-dimensional non-metric multidimensional scaling (NMDS) using Bray-Curtis dissimilarity of roots taken in summer and autumn after planting or the following spring at Ellerhoop. Vectors represent the correlation coefficient between the corresponding genus and the NMDS score. Relative lengths and the directions of the vectors indicate the influence of the respective genera (RA > 0.5%). The third axis is not shown. Results of the one-way analysis of similarities are shown at the table above, significant differences are highlighted in bold (*p* ≤ 0.05).

Supplementary Table 1 Origins of the different isolates. Isolates were obtained from roots grown only in ARD plots (yellow), grass plots (green) or both plots (gray). Isolates were identified using Sanger sequencing and blasting against the NCBI database. The closest hit at species level is shown.

Supplementary Table 2 Isolates obtained in the culture independent approach blasted against ASVs from amplicon sequencing (culture independent approach). Shown is the isolate identified by Sanger sequencing (closest species hit at NCBI database) and the corresponding ASV, which showed the highest Blast score in a local Blastn. The identity shows the matching bp of the isolate (query) divided by the bp of the matching ASV (sbjct) in percentage. The mean relative abundance (average over the three sampling times) of these ASVs in roots grown in ARD and grass soils from Heidgraben (H) and Ellerhoop (E) is shown.
