# Supplementary figures and images for: Dynamics of Bacterial Root Endophytes of Malus domestica Plants Grown in Field Soils Affected by Apple Replant Disease
Source: Front Microbiol. 2022 Mar 25;13:841558. doi: 10.3389/fmicb.2022.841558 (PMC8993231; doi:10.3389/fmicb.2022.841558)

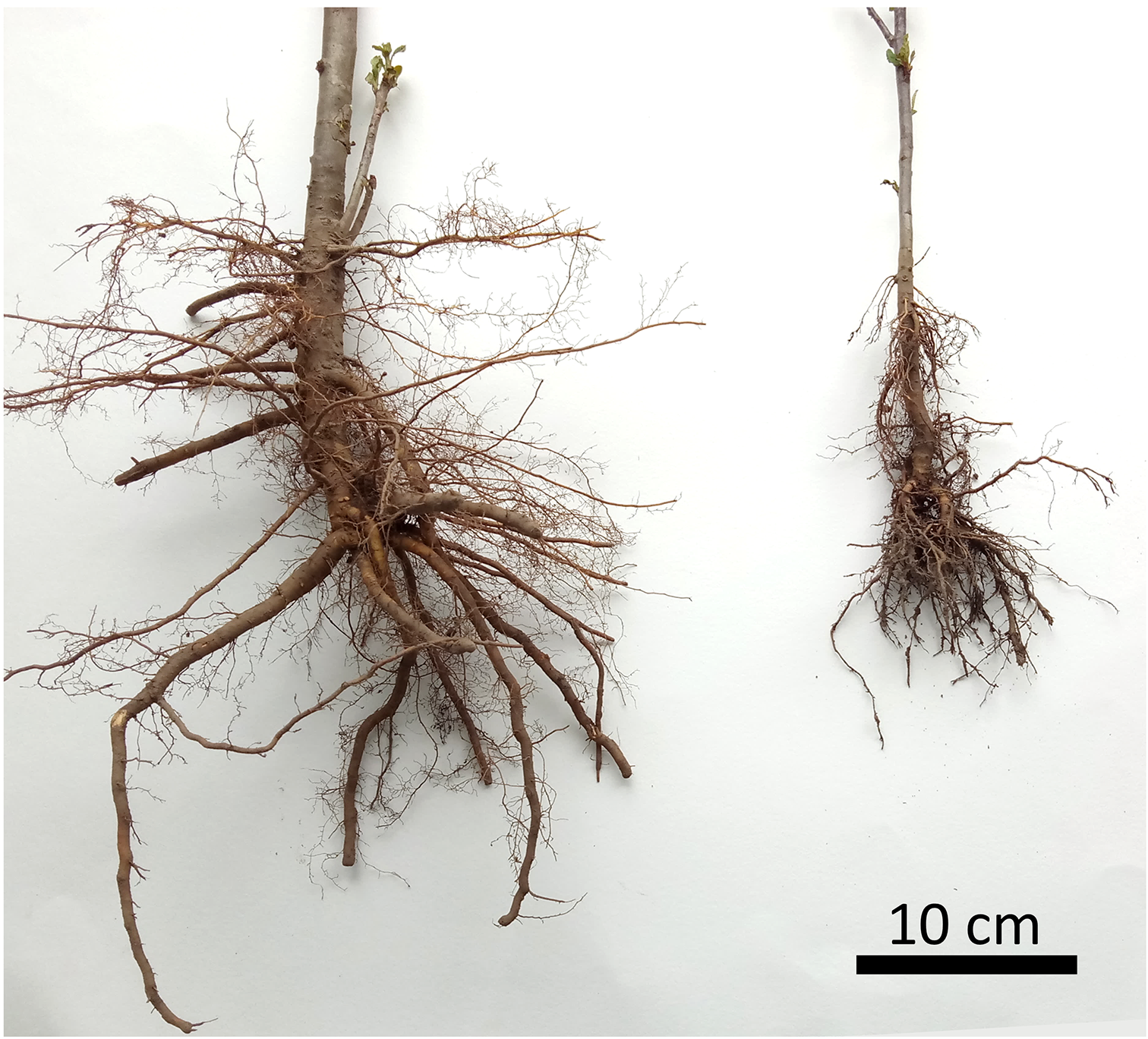

Supplement: Supplementary Figure 1 — Apple roots in spring after 12 months of growth in grass soil (left) or ARD soil (right) at Ellerhoop (photo: Alicia Balbín-Suárez). [file Image_1.TIF]

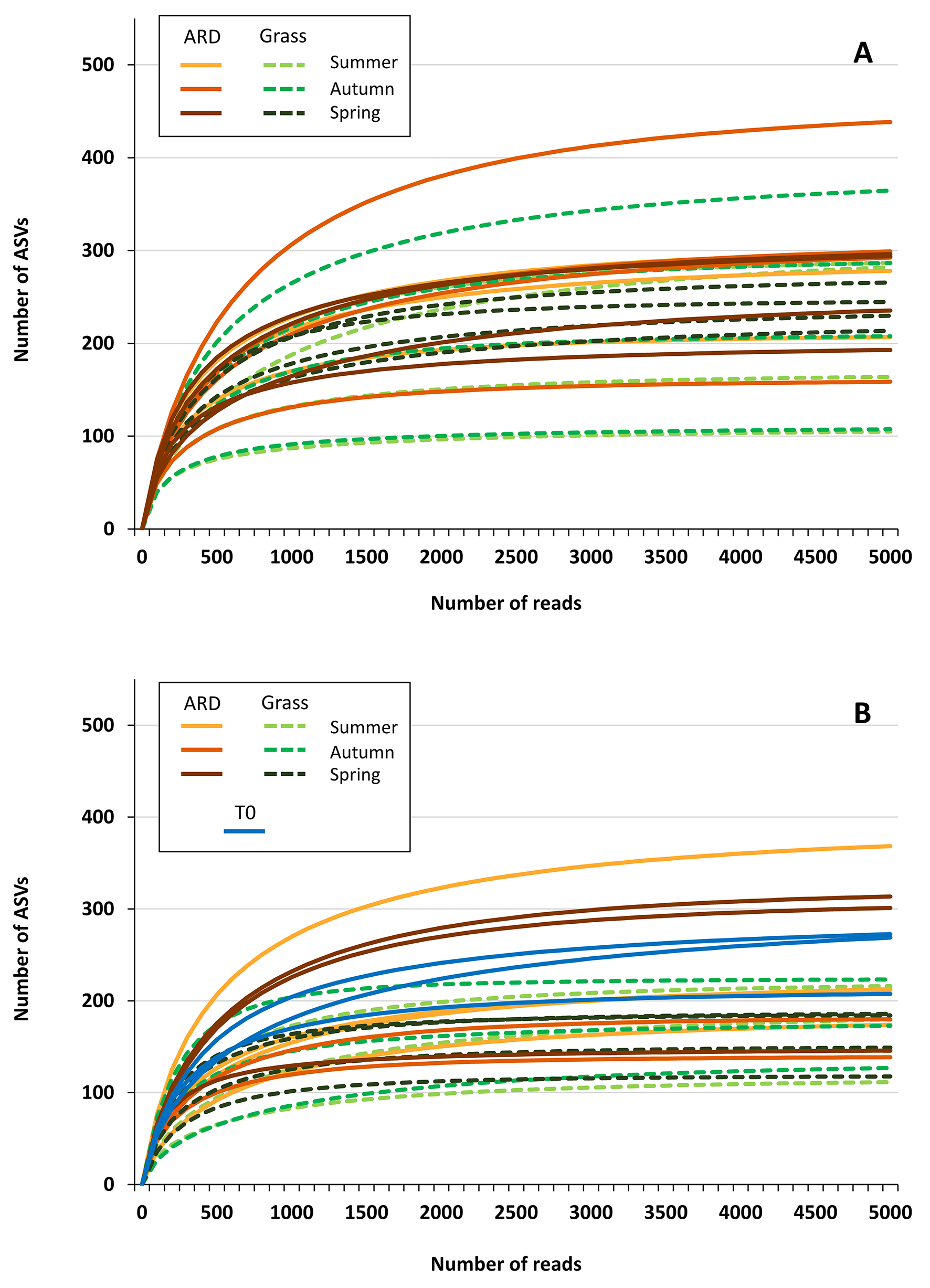

Supplement: Supplementary Figure 2 — Rarefaction curves showing the number of observed ASVs in all samples of Bittenfelder roots grown in ARD or grass soil at Heidgraben (A) and Ellerhoop (B) and taken in 3 months (summer), 7 months (autumn) or 12 months after planting (spring). Each line represents one sample of Bittenfelder roots taken in summer, autumn or spring. All samples were rarefied at 4,213 reads. [file Image_2.TIF]

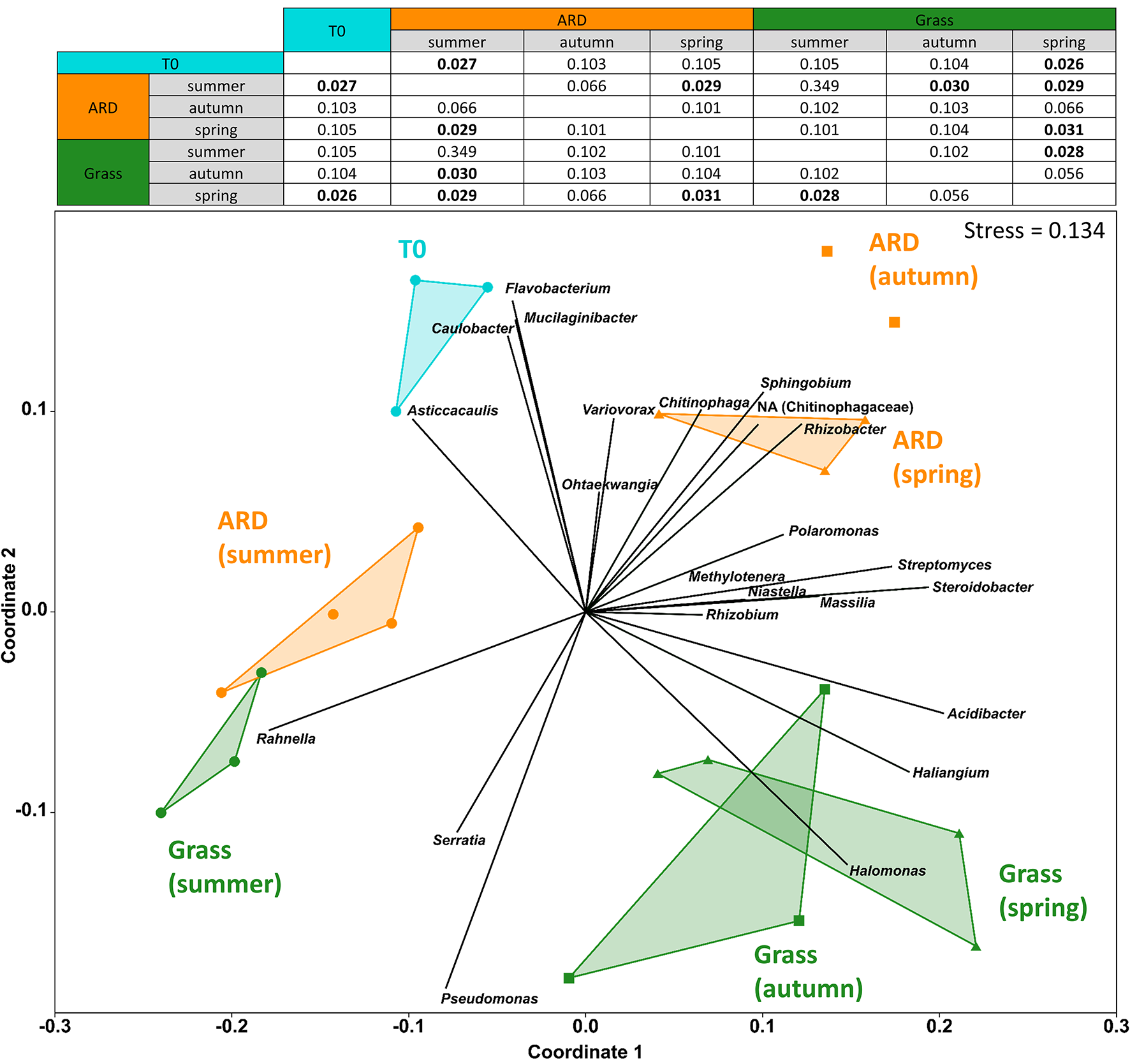

Supplement: Supplementary Figure 3 — Three-dimensional non-metric multidimensional scaling (NMDS) using Bray-Curtis dissimilarity of roots taken in summer and autumn after planting or the following spring at Ellerhoop. Vectors represent the correlation coefficient between the corresponding genus and the NMDS score. Relative lengths and the directions of the vectors indicate the influence of the respective genera (RA > 0.5%). The third axis is not shown. Results of the one-way analysis of similarities are shown at the table above, significant differences are highlighted in bold (p ≤ 0.05). [file Image_3.TIF]

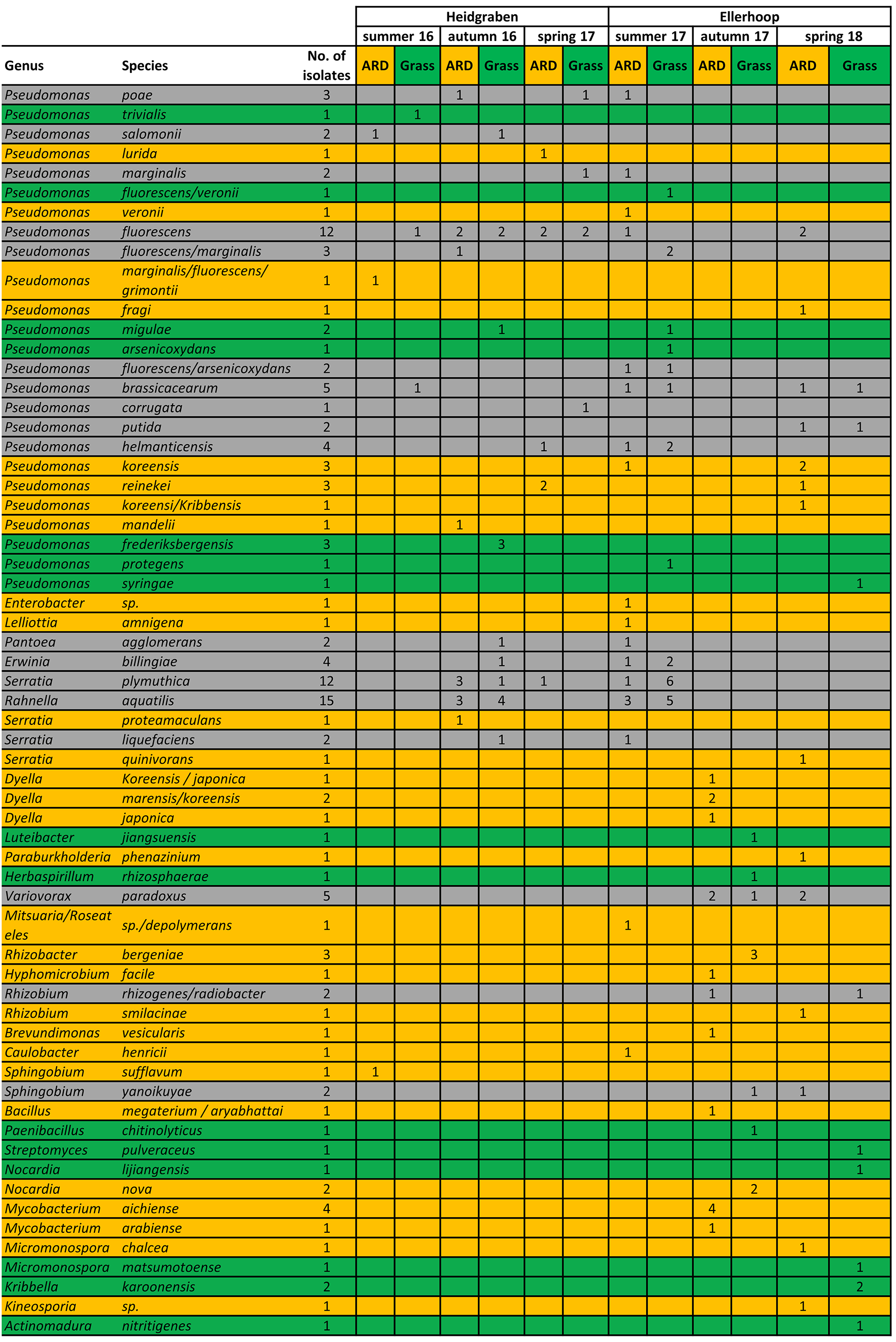

Supplement: Supplementary Table 1 — Origins of the different isolates. Isolates were obtained from roots grown only in ARD plots (yellow), grass plots (green) or both plots (gray). Isolates were identified using Sanger sequencing and blasting against the NCBI database. The closest hit at species level is shown. [file Data_Sheet_1.zip › Table_1.tif]

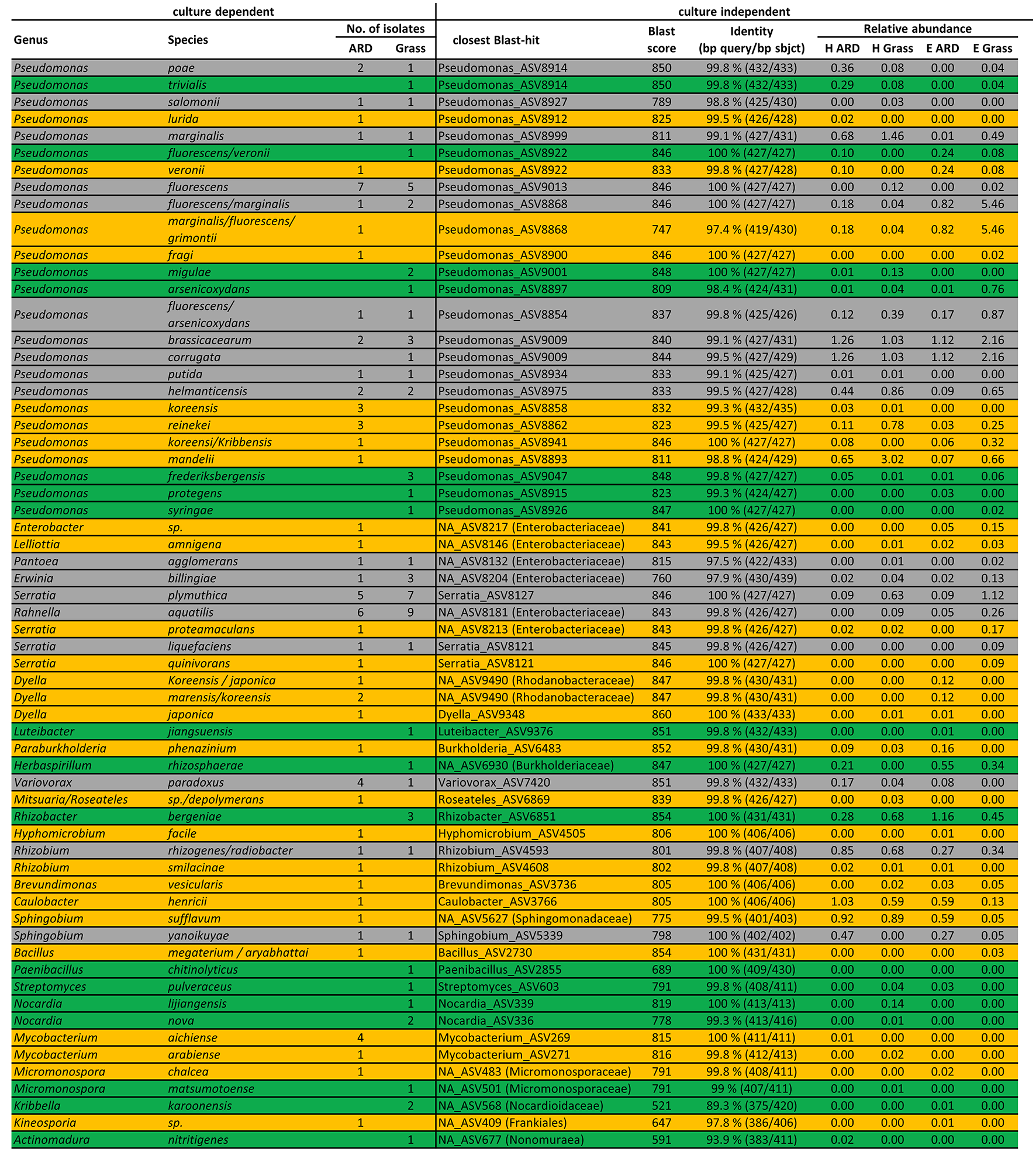

Supplement: Supplementary Table 1 — Origins of the different isolates. Isolates were obtained from roots grown only in ARD plots (yellow), grass plots (green) or both plots (gray). Isolates were identified using Sanger sequencing and blasting against the NCBI database. The closest hit at species level is shown. [file Data_Sheet_1.zip › Table_2.tif]
